# Differential pathology of P102L-associated Gerstmann–Stäussler–Scheinker disease: exclusive presence of 8-kDa protease-resistant prion protein vs. co-existence of 8-kDa and type-1 protease-resistant prion protein, with a focus on codon 129 polymorphism

**DOI:** 10.1080/19336896.2025.2560823

**Published:** 2025-09-16

**Authors:** Hideko Noguchi, Motoi Yoshimura, Akihiro Watanabe, Sachiko Koyama, Naonori Sakurada, Masahiro Shijo, Takaaki Kanemaru, Keita Kai, Shinichi Aishima, Haruki Koike, Yoshio Tsuboi, Naokazu Sasagasako, Hiroyuki Honda

**Affiliations:** a Department of Neuropathology, Graduate School of Medical Sciences, Kyushu University, Fukuoka, Japan; Division of Neurology, Department of Neurology, Neuro Muscular Center, b National Hospital Organization, Omuta Hospital, Omuta, Japan; c Neuropathology Center, National Hospital Organization, Omuta Hospital, Omuta, Japan; d Division of Respirology, Neurology and Rheumatology, Department of Medicine, Kurume University School of Medicine, Kurume, Japan; e Department of Neurology, Kyushu Central Hospital of the Mutual Aid Association of Public School Teachers, Fukuoka, Japan; f Department of Morphology Core Unit, Kyushu University Hospital, Fukuoka, Japan; g Department of Pathology, Saga University Hospital, Saga, Japan; h Department of Scientific Pathology Graduate School of Medical Sciences, Kyushu University, Fukuoka, Japan; i Division of Neurology, Department of Internal Medicine, Faculty of Medicine, Saga University, Saga, Japan; j Department of Neurology, Faculty of Medicine, Fukuoka University, Fukuoka, Japan

**Keywords:** Amyloid, cotton wool, Gerstmann–Sträussler–Scheinker disease, prion diseases, spongiform degeneration

## Abstract

Gerstmann – Sträussler – Scheinker disease (GSS) is a hereditary prion disease characterized by clinicopathological heterogeneity. In Japan, the most common mutation is P102L, typically associated with prion protein (PrP) plaques, spongiform changes, and synaptic PrP deposits. Two major protease-resistant PrP (PrP^res^) types occur: type-1 PrP^res^ (21 kDa) and 8-kDa PrP^res^. The *PRNP* codon 129 polymorphism (methionine or valine) influences disease phenotype, but factors underlying exclusive 8-kDa PrP^res^ expression remain unclear. We analysed two sibling P102L GSS cases exclusively exhibiting 8-kDa PrP^res^ (Case 1: 129 MM, haplotype: P102L–129 M, treated with pentosan polysulfate; Case 2: 129 MV, haplotype: P102L–129 M, treated with quinacrine hydrochloride and quinine hydrochloride) and four P102L–129 MM GSS cases exhibiting type-1 and 8-kDa PrP^res^ (Cases 3–6) to elucidate the clinicopathological effect of 8-kDa PrP^res^ and *PRNP* codon 129 polymorphisms. Case 1 predominantly exhibited amyloidogenic PrP plaques; Case 2 exhibited non-amyloidogenic cotton-wool PrP plaques, with minimal synaptic PrP deposits. Despite prolonged survival ( > 20 years), spongiform degeneration and neuronal loss were mild. Cases 3–6 showed numerous amyloidogenic PrP plaques, moderate-to-severe synaptic PrP deposits, and significant tissue damage. Homoeostatic microglial markers were preserved in Cases 1 and 2 but absent in Cases 3–6. Cotton-wool PrP plaques lacked amyloid cores and were associated with 8-kDa PrP^res^ and codon 129 V from the normal allele. Tissue damage was mild in P102L GSS cases exhibiting 8-kDa PrP^res^, suggesting lower pathogenicity. Cotton-wool PrP plaque formation likely involves 8-kDa PrP^res^ and codon 129 V. Further large-scale studies are warranted to elucidate these mechanisms.

## Introduction

Human prion diseases, including sporadic Creutzfeldt – Jakob disease (sCJD), Gerstmann – Stäussler – Scheinker disease (GSS), variant CJD, and iatrogenic CJD, are fatal neurodegenerative disorders [1]. Prion disease pathogenesis involves the post-translational conversion of the cellular prion protein (PrP^c^) to an abnormal pathogenic prion protein (PrP) (scrapie PrP [PrP^Sc^]) [1]. PrP^Sc^ has a high β-sheet content that makes it partially resistant to proteinase K (PK) digestion (protease-resistant PrP: PrP^res^) [2]. sCJD is characterized by rapidly progressive dementia and myoclonus, which is pathologically identified with spongiform degeneration, neuronal loss, and synaptic PrP deposits [3]. PrP^res^ in sCJD was characterized by three major bands corresponding to unglycosylated, monoglycosylated, and diglycosylated forms. Additionally, two isoforms of PrP^res^ are distinguished based on the molecular weight of their unglycosylated forms: type-1 PrP^res^ (unglycosylated form 21 kDa) and type-2 PrP^res^ (unglycosylated form 19 kDa) [3,4]. Furthermore, a polymorphism at codon 129 of *PRNP* (methionine [M] or valine [V]), in combination with the PrP^res^ isoform, might contribute to the clinicopathological heterogeneity of sCJD. GSS is an autosomal dominantly inherited prion disease [5]. Gene mutations known to cause GSS include P102L, P105L, A117V, G131V, F198S, and Q217R [6]. In Japan, the most common mutation causing GSS is P102L (proline to leucine), which is the second most frequent mutation in hereditary prion diseases [7]. The clinical manifestations of P102L GSS are heterogeneous and include cerebellar ataxia, sensory disturbances, and slowly progressive dementia [1]. Phenotypic variability has been observed among individuals with the same genetic mutation or within the same family [8–14]. Pathologically, GSS is characterized primarily by amyloidogenic PrP plaques in the cerebral cortex, cerebellar cortex, and basal ganglia, often accompanied by synaptic PrP deposits and spongiform degeneration [6].

Immunoblot analysis of brain homogenates from P102L GSS revealed two major PrP^res^ patterns: one with a molecular mass of 21 kDa, similar to the type-1 PrP^res^ observed in sCJD, and the other with a low molecular mass of 8-kDa PrP^res^ [15]. Both PrP^res^ types were observed; however, some cases exhibited only 8-kDa low-molecular-weight PrP^res^. In GSS, as in sCJD, a genotype – phenotype relationship associated with the genetic polymorphism of *PRNP* codon 129 involving M and V has been suggested [16–18]. Bianca et al. reported that a patient with GSS carrying the P102L-129 MV mutation (haplotype P102L-129 V) exhibited prominent psychiatric features, including apathy and depression, without any cerebellar signs [17]. Hill et al. reported one case of P102L-129 MV (haplotype: P102L-129 M) that exhibited only 8-kDa PrP^res^ [18]. Young et al. described the clinicopathological findings of a case with the P102L-129VV mutation (haplotype: P102L-129 V). Clinically, the patient was presented with seizures as an initial symptom and exhibited no signs of dementia. Pathologically, amyloid PrP plaques were observed, but spongiform degeneration was absent [16]. Genetic mutations and codon 129 polymorphism in GSS, and their associated clinicopathological heterogeneity, have been reported; however, these findings remain limited. Moreover, the precise mechanisms underlying the presence of PrP plaques alone in some cases, compared with PrP plaques and synaptic PrP deposits in others, and the presence of 8-kDa PrP^res^ in some instances compared with the co-existence of 8- and 21-kDa PrP^res^ in others, remain subjects of ongoing investigation and debate.

We conducted a pathological examination of two sibling cases of P102L GSS (Cases 1 and 2) exhibiting only 8-kDa PrP^res^, and four cases of P102L GSS (Cases 3 to 6) exhibiting type-1 PrP^res^ and 8-kDa PrP^res^. Case 1 was identified as P102L-129 MM (haplotype: P102L-129 M), and had a history of treatment with pentosan polysulfate. Case 2 was identified as P102L-129 MV, with the normal allele carrying 129 V (haplotype: P102L-129 M), and had been treated with quinacrine hydrochloride and quinine hydrochloride. Case 1 showed numerous amyloidogenic PrP plaques but only minimal synaptic PrP deposits. In contrast, Case 2 exhibited a unique pattern of non-amyloidogenic PrP plaques with only minimal synaptic PrP deposits. These non-amyloidogenic PrP plaques morphologically resembled the cotton-wool plaques composed of amyloid-β described by Crook et al. in familial Alzheimer’s disease with presenilin-1 mutations [19]. With reference to this report, the non-amyloidogenic PrP plaques observed in Case 2 are referred to as cotton-wool PrP plaques. Cases 3–6 had a prion genotype of P102L-129 MM (haplotype: P102L-129 M) and demonstrated numerous amyloidogenic PrP plaques along with moderate-to-severe synaptic PrP deposits. In this study, through a clinicopathological investigation of these cases, we examined the relationships between PrP gene mutations, codon 129 polymorphism, haplotype, 8-kDa low-molecular-weight PrP^res^, PrP plaque formation (amyloidogenic/non-amyloidogenic), and histopathological features, specifically addressing the involvement of resident microglia.

## Results

**Figure 1.**
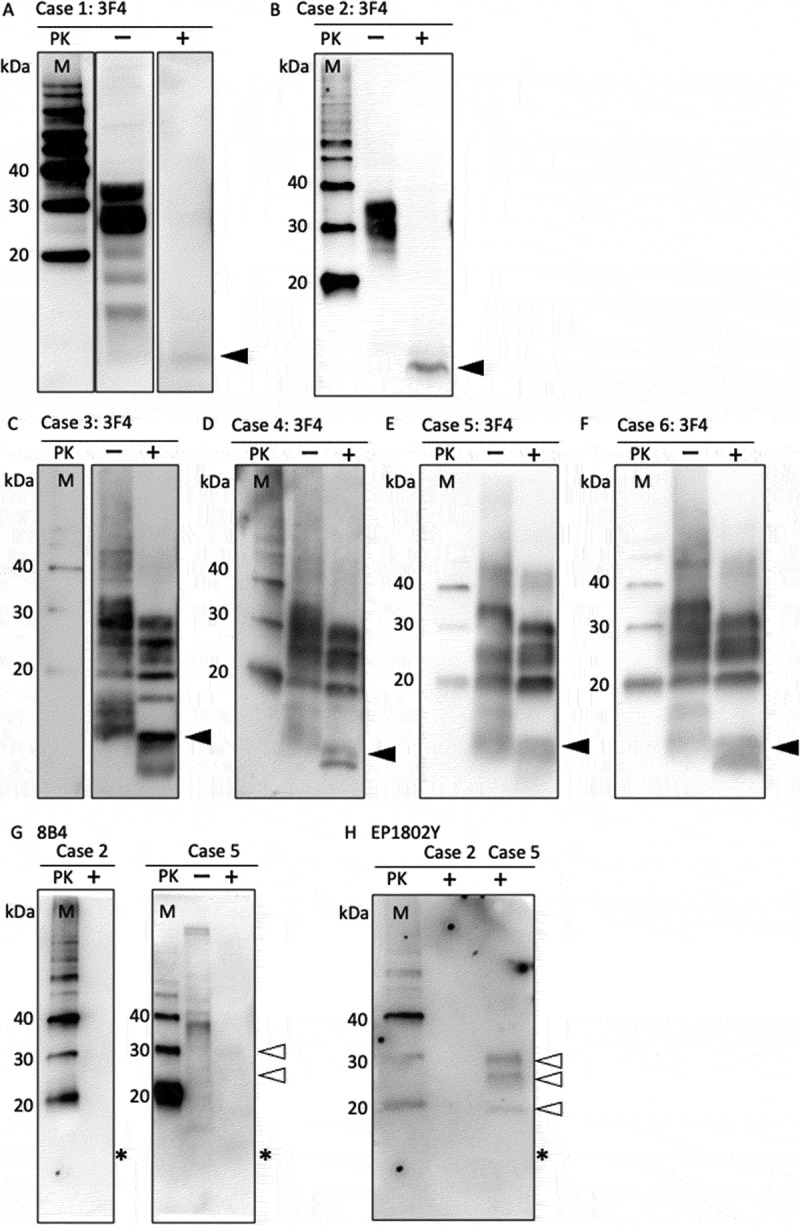
Western blot analysis of prion protein in the frontal cortex using anti-prion protein antibody (3F4: A-F, 8B4: G, EP1802Y: H). (A, B) Only 8-kDa PrP^res^ (arrowheads) was detected, with no evidence of type-1 PrP^res^. (C-F) 8-kDa PrP^res^ (arrowheads) and type-1 PrP^res^ ranging from 21-kDa unglycosylated PrP were detected. PrP, prion protein; PrP^res^, protease-resistant prion protein. (G) Using the N-terminal PrP antibody 8B4, 8-kDa PrP^res^ was not detected (asterisk) in either Case 2 or Case 5. In Case 5, faint signals of mono-glycosylated and di-glycosylated type 1 PrP^res^ were identified (white arrowheads). (H) Using the C-terminal PrP antibody EP1802Y, the 8-kDa PrP^res^ signal was not identified in either Case 2 or Case 5 (asterisk). In contrast, three bands of type 1 PrP^res^ were observed in Case 5 (white arrowheads).

Table 1 shows the examined cases. PK-untreated samples from the frontal cortex of Patients 1 and 2 exhibited strong PrP signals, which included non-glycosylated, monoglycosylated, and diglycosylated PrP forms ranging from approximately 25 to 35 kDa (Figure 1(A,B)). PK-treated samples from both cases displayed only an 8-kDa PrP^res^ signal, lacking the typical type-1 PrP^res^ three-band pattern (Figure 1(A,B): arrowheads). In contrast, PK-treated samples from Cases 3 to 6 showed a typical type-1 PrP^res^ three-band pattern and an 8-kDa PrP^res^ signal (Figure 1(C–F): arrowheads). Western blot analysis with the N-terminal PrP antibody 8B4 did not reveal an 8-kDa PrP^res^ signal in either Case 2 or Case 5 (Figure 1G: asterisk). By contrast, in Case 5, faint signals corresponding to the mono- and diglycosylated forms of type 1 PrP^res^ were detected (Figure 1(G), white arrowheads). The C-terminal PrP antibody EP1802Y likewise failed to detect 8-kDa PrP^res^ in either Case 2 or Case 5 (Figure 1(H): asterisk). In Case 5, however, three distinct bands corresponding to type 1 PrP^res^ were observed (Figure 1(H), white arrowheads).

**Table 1.** Profiles of the cases examined in this study.

Case No.	Diagnosis	Codon 129	haplotype	PrPres type	AD (y)	Sex	DD (y)	Clinical phenotype	Initial symptoms	Symptoms	Therapy	References
1*	GSS (P102L)	M/M	P102L-129 M	8kDa	70	F	20	Classical GSS	Cerebellar ataxia	Dementia, Akinetic mutism	pentosan polysulfate (50y)	[22] case 8
2*	GSS (P102L)	M/V	P102L-129 M	8kDa	78	F	25	Dementia	Dementia	Cerebellar ataxia, Akinetic mutism	quine (59y), quinacrine (59y)	
3	GSS (P102L)	M/M	P102L-129 M	type1 + 8kDa	79	F	5	Classical GSS	Cerebellar ataxia	Dementia, Akinetic mutism	no	[9] case 3
4	GSS (P102L)	M/M	P102L-129 M	type1 + 8kDa	70	M	10	Classical GSS	Cerebellar ataxia	Dementia, Akinetic mutism, Myoclonus	no	[9] case 4
5	GSS (P102L)	M/M	P102L-129 M	type1 + 8kDa	78	F	13	Classical GSS	Cerebellar ataxia	Dementia, Akinetic mutism	no	[9] case 5
6	GSS (P102L)	M/M	P102L-129 M	type1 + 8kDa	65	M	5	Dementia	Dementia	Cerebellar ataxia, Akinetic mutism	no	[9] case 6

*, sisters; AD, age at death; DD, disease duration; F, female; kDa, kilodalton; L, leucine; M, male; M/M, methionine/methionine; M/V, methionine/valine; No, number; P, proline; PrPres, protease resistant prion protein; y, years.

**Figure 2.**
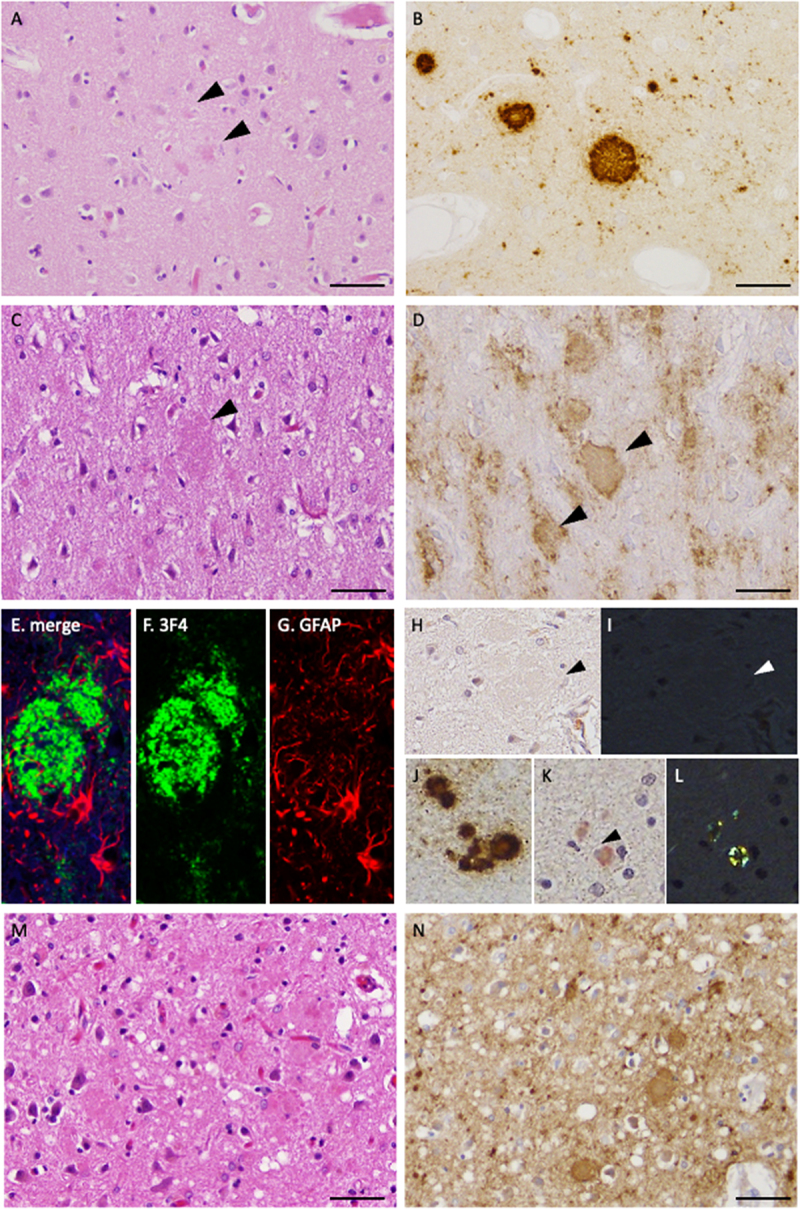
Histopathological findings in the frontal cortex of GSS cases (A, B: case 1, C – L: case 2, M, N: case 3). (A) Hematoxylin and eosin staining revealed prion protein (PrP) plaques (arrowheads) and mild spongiform changes. Neuronal loss was mild. (B) Immunohistochemistry using the PrP antibody (3F4) revealed PrP plaques and mild synaptic PrP deposits. (C) hematoxylin and eosin staining revealed cotton-wool-like plaques (arrowhead). Spongiform changes and neuronal loss were mild. (D) Immunostaining with 3F4 revealed cotton-wool PrP plaques (arrowheads) and mild synaptic PrP deposits. (E – G) Double immunofluorescence staining with 3F4 (red) and GFAP (green) revealed small spheroidal clusters of PrP, referred to here as cotton-wool PrP plaques, along with the cytoplasm and processes of surrounding reactive astrocytes. (H, I) Cotton-wool PrP plaques did not exhibit staining with Congo red (H: arrowhead). Green birefringence was not observed under polarized light (I: arrowhead). (J – L) Immunostaining with 3F4 demonstrated PrP plaques in the cerebellar cortex (J). The PrP plaques were positive for Congo red staining (K: arrowhead), and green birefringence was observed under polarized light (L). GSS, Gerstmann–Stäussler–Scheinker disease; PrP, prion protein.

Table 2 shows the summary of the pathological findings. In Case 1, the frontal cortex exhibited mild spongiosis and neuronal loss (Figure 2(A)). PrP plaques were also frequently observed (Figure 2(A), arrowheads). Immunohistochemistry revealed numerous PrP plaques and mild synaptic PrP deposits (Figure 2(B)). Table 3 presents the extent of PrP deposition, spongiform degeneration, and neuronal loss in the cerebrum, cerebellum, and brainstem. In summary, moderate neuronal loss was observed in the putamen and thalamus. Numerous PrP plaques were present in the putamen and thalamus, along with severe synaptic PrP deposits. PrP plaques are predominant over synaptic PrP deposits in the cerebrum, cerebellum, and brainstem. In Case 2, the frontal cortex showed mild-to-moderate spongiosis and neuronal loss (Figure 2(C)). Cotton-wool-like structures were also observed (Figure 2(C), arrowhead). Immunohistochemistry for PrP revealed numerous cotton-wool-like PrP plaques (cotton-wool PrP plaques) and mild-to-moderate synaptic PrP deposits (Figure 2(D); cotton-wool PrP plaques: arrowheads). Double immunofluorescence for PrP and GFAP demonstrated that the cotton-wool PrP plaques were surrounded by GFAP-positive astrocytes (Figure 2(E-G)). Unlike the typical PrP plaques observed in GSS, cotton-wool PrP plaques showed no distinct cores. Additionally, the cotton-wool PrP plaques were not stained with Congo red (Figure 2(H), I: arrowhead). However, in limited areas such as the cerebellum and amygdala, PrP plaques with amyloidogenic cores were occasionally observed (Figure 2(J-L), J: PrP immunostaining, K, L: Congo red staining). However, these amyloidogenic PrP plaques appeared smaller than those typically observed in conventional P102L GSS. Synaptic PrP deposits and PrP plaques, which are seen in dura graft CJD, were not present at the dura graft site. Table 3 presents the details of the pathological findings. In summary, in the cerebrum, cotton-wool PrP plaques predominated in many areas, with synaptic PrP deposits ranging from mild to moderate. Synaptic PrP deposits are predominant in the brainstem. Spongiosis was extremely pronounced in the putamen, globus pallidus, and thalamus, rendering the identification of PrP deposits impossible. In Cases 3–6, numerous PrP plaques and moderate-to-severe synaptic PrP deposits were observed (Figure 2(M,N)). Table 3 presents the details of the pathological findings.

**Table 2.** Pathological findings including prion protein deposits and microglia/macrophages in the fronta cortex.

			Frontal cortex				
Case No.	PMI (H)	BW (g)	PrP plaque	synaptic PrP	spongiform change	neuronal loss	microglia/macrophage
1	3	1055	amyloidgenic	mild	mild	mild	Most regident microglia were ramified with fine processes, indicating homoeostatic state. TMEM119 were preserved but P2RY12 were diminished.
2	12.5	730	non amyloidgenic cotton wool-like plaque	mild-moderate	mild-moderate	mild	Most regident microglia were ramified with fine processes, indicating homoeostatic state. TMEM119 were preserved but P2RY12 were diminished.
3	2.5	1200	amyloidgenic	moderate	moderate	mild-moderate	Many activated microglia/macrophageswere in neuropil and around PrP plaques.TMEM119 and P2RY12 were mostly diminished and were present only at PrP plaques.
4	69	1010	amyloidgenic	moderate	moderate	moderate-severe	Many activated microglia/macrophages were in neuropil and around PrP plaques. TMEM119 and P2RY12 were mostly diminished and were present only at PrP plaques.
5	7	1020	amyloidgenic	severe	moderate	moderate	Many activated microglia/macrophages were in neuropil and around PrP Plaques. TMEM119 and P2RY12 were mostly diminished and were present only at PrP plaques.
6	2	820	amyloidgenic	severe	severe	severe	Iba1 and HLA-DR-positive activated microglia/macro phages increased more than other GSS cases. TMEM119 and P2RY12 were mostly diminished and were present only at PrP plaques.

BW, brain weight; g, gram; H, hours; No, number; PMI, post mortem interval; PrP, prion protein.

**Table 3.** Pathological findings of prion protein deposition and neuronal degeneration by brain region.

Case No.		Frontal cortex	Temporal cortex	Putamen	Globus pallidus	Thalamus	Hippocampus	Cerebellum	Midbrain	Pons	Medullo oblongata
1	spongiosis/neuronal loss	1/1	1/1	1/2	1/1	2/2	1/1	2/1	1/1	1/1	1/1
	PrP deposits (AP/nonAP/Sy)	3/0/1	3/0/1	3/0/3	1/0/1	3/0/3	2/0/1	3/0/1	0/0/1	0/0/1	0/0/1
2	spongiosis/neuronal loss	1–2/1–2	1–2/1–2	3/3	3/3	3/3	2/2	2/2	1/1	1/1	1/1
	PrP deposits (AP/nonAP/Sy)	0/3/1–2	0/3/1–2	0/0/1	0/0/1	0/0/1	0/2/1	2/2/1–2	0/0/1–2	0/0/1–2	0/0/1–2
3	spongiosis/neuronal loss	2/2	2/2	2/2	2/2	3/3	1/1	2/2	1/1	1/1	1/1
	PrP deposits (AP/nonAP/Sy)	3/0/3	3/0/2	3/0/2	3/0/1	3/0/1	1/0/1	2/0/1	1/0/2	1/0/1	0/0/1
4	spongiosis/neuronal loss	2/3	2/3	2/2	3/3	3/2	2/1	2/2	2/1	0/1	1/1
	PrP deposits (AP/nonAP/Sy)	3/0/2	3/0/2	3/0/2	3/0/1	2/0/1	2/0/1	2/0/1	1/0/1	1/0/1	0/0/2
5	spongiosis/neuronal loss	2/2	3/2	2/2	2/3	2/2	1/1	2/2	2/2	1/1	1/1
	PrP deposits (AP/nonAP/Sy)	3/0/3	3/0/3	3/0/3	3/0/2	2/0/3	2/0/1	3/0/2	1/0/2	1/0/2	0/0/2
6	spongiosis/neuronal loss	3/3	3/3	1/2	1/2	1/2	1/1	2/2	2/2	2/2	1/1
	PrP deposits (AP/nonAP/Sy)	3/0/3	3/0/3	3/0/3	3/0/2	3/0/2	2/0/2	3/0/3	2/0/3	2/0/2	1/0/2

0, none; 1, mild; 2, moderate; 3, severe; AP, amyloidogenic plaque; nonAP, non amyloidogenic plaque; PrP, prion protein; Sy, synaptic prion protein deposits.

In summary, in Cases 1 and 2, amyloidogenic PrP plaques and non-amyloidogenic cotton-wool PrP plaques were predominant over synaptic PrP deposits. Additionally, spongiform changes and neuronal loss were limited to mild-to-moderate levels. In contrast, in Cases 3–6, amyloidogenic PrP plaques were observed in large amounts, and synaptic PrP deposits ranged from moderate to severe. Furthermore, the spongiform changes and neuronal loss were moderate to severe.

**Figure 3.**
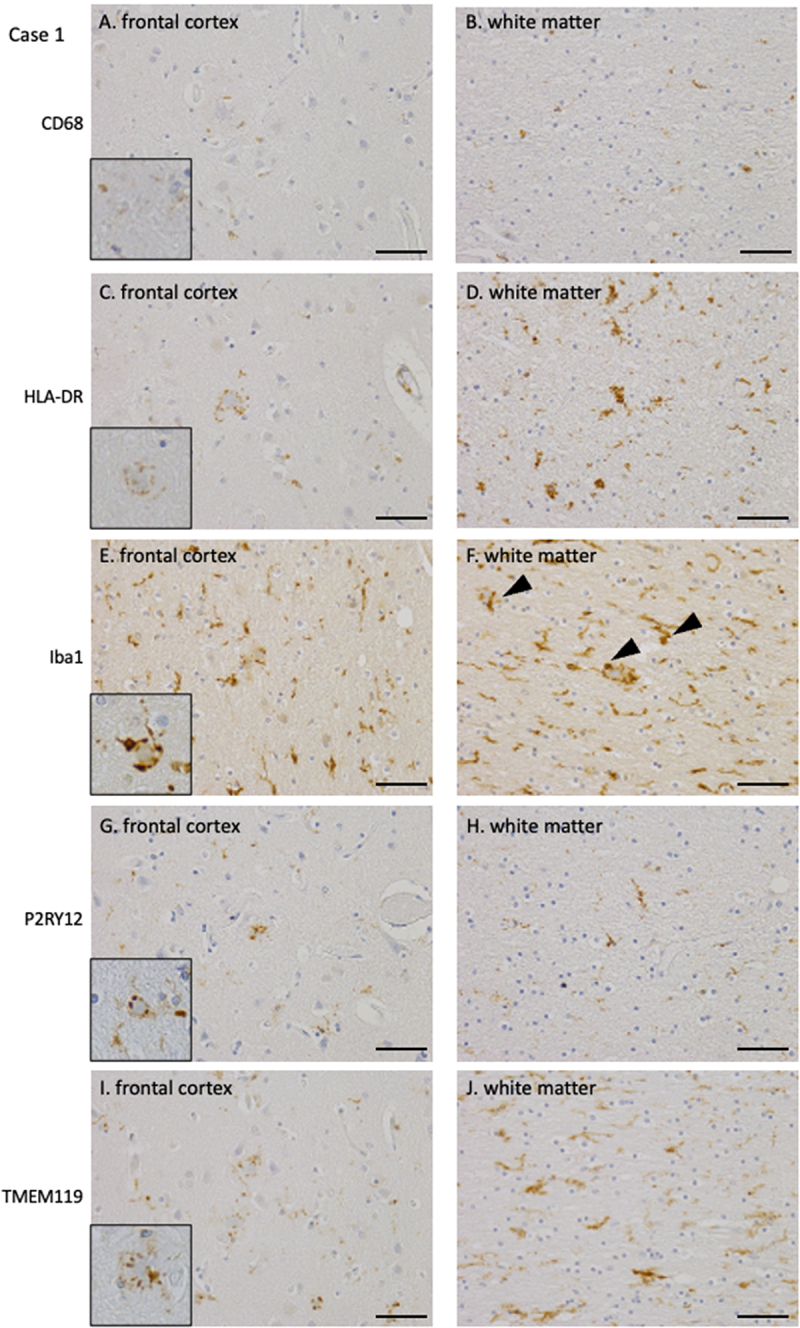
Immunohistochemical staining of microglia/macrophages in the frontal cortex and white matter of Case 1 (A, B: CD68, C, D: HLA-DR, E, F: Iba1, G, H: P2RY12, I, J: TMEM119, A, C, E, G, I: cortex, B, D, F, H, J: white matter) (A, B) CD68 immunohistochemistry demonstrated minimal positive macrophages in the cortical and white matter regions. (C, D) HLA-DR immunohistochemistry identifies a sparse presence of positive macrophages surrounding PrP plaques in the cortex and a small number of macrophages in the white matter. (E, F) Iba1 immunohistochemistry revealed positive microglia surrounding PrP plaques and within the neuropil in the cortex, and a mild increase in microglia in the white matter. (G, H) P2RY12 immunohistochemistry revealed a small number of positive microglia around PrP plaques and within the neuropil in the cortex, and a small number in the white matter. (I, J) TMEM119 immunohistochemistry revealed a small number of positive microglia around PrP plaques and within the neuropil in the cortex, whereas a relatively larger number of positive microglia remained in the white matter. PrP, prion protein.

**Figure 4.**
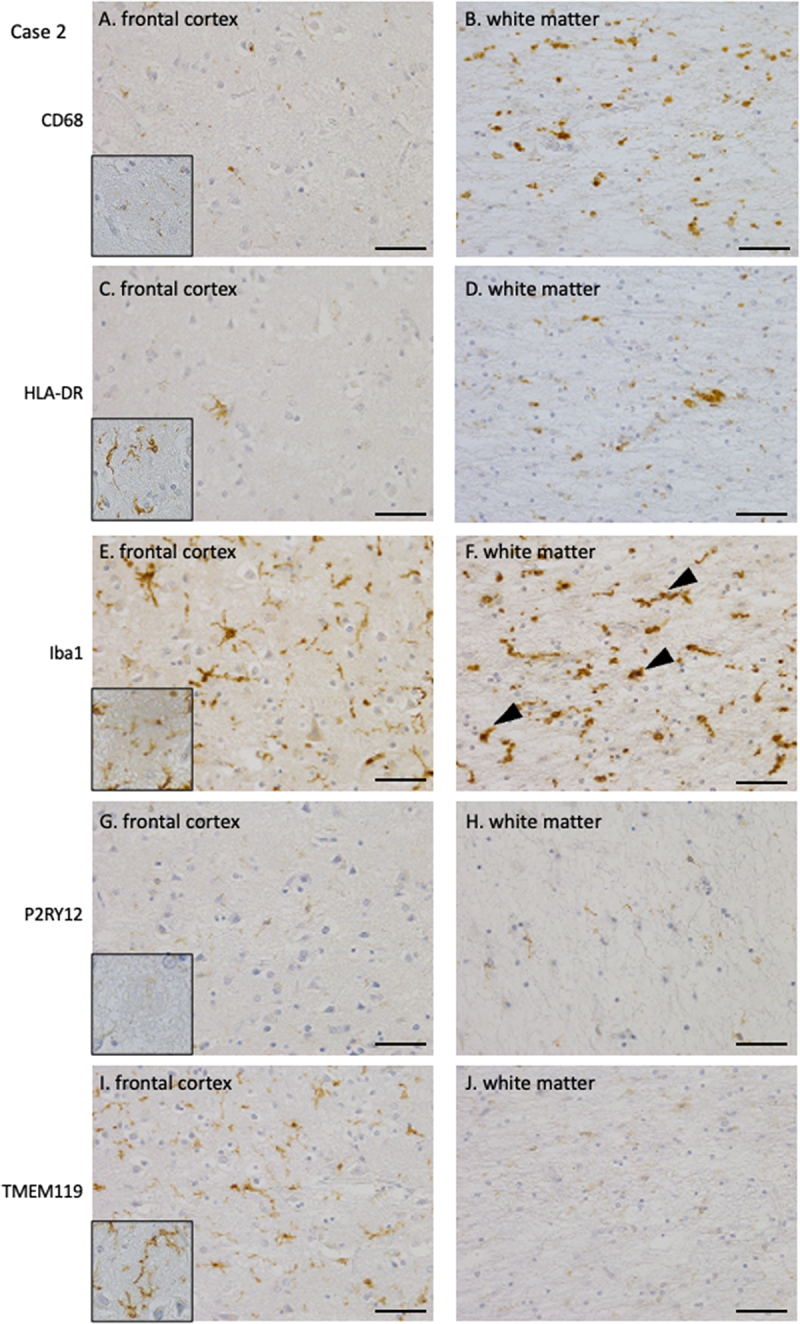
Immunohistochemical staining of microglia/macrophages in the frontal cortex and white matter of Case 2 (A, B: CD68, C, D: HLA-DR, E, F: Iba1, G, H: P2RY12, I, J: TMEM119, A, C, E, G, I: cortex, B, D, F, H, J: white matter) (A, B) CD68 immunohistochemistry revealed a few positive macrophages in the cortex and a modest number of activated macrophages in the white matter. (C, D) HLA-DR immunohistochemistry demonstrated a sparse presence of positive cells around PrP plaques in the cortex and a limited number in the white matter. (E, F) Iba1 immunohistochemistry revealed positive microglia surrounding PrP plaques and within the neuropil in the cortex, and a mild increase in microglia in the white matter. (G, H) P2RY12 immunohistochemistry demonstrated positive microglia around PrP plaques, with only a sparse presence observed in the cortical neuropil and white matter. (I, J) TMEM119 immunohistochemistry demonstrated a small number of positive microglia around PrP plaques and within the neuropil in the cortex, whereas only a sparse presence was observed in the white matter. PrP, prion protein.

The degree of tissue damage in each case was examined, with a focus on microglial and macrophage activation. Table 2 summarizes the pathological findings. In Case 1, CD68- and HLA-DR-positive activated macrophages were rarely observed (Figure 3(A–C)). HLA-DR-positive microglia were only observed in small numbers around PrP plaques (Figure 3C, inset) and in a limited area of the white matter (Figure 3(D)). A moderate amount of Iba1-positive microglia were observed in the cortex; however, no significant increase was observed, and most of the microglia were ramified in appearance. Iba1-positive microglia were often observed around the PrP plaques (Figure 3(D), inset). In contrast, in the white matter, Iba1-positive microglia with enlarged cytoplasm and thick processes were occasionally observed (Figure 3(F): arrowheads). P2RY12- and TMEM119-positive microglia, markers of resident microglia indicating a homoeostatic state, remained around PrP plaques and in small amounts within the neuropil of the cortex (Figure 3(G), I, insets: PrP plaques). In the white matter, microglia in a homoeostatic state positive for P2RY12 and TMEM119 were preserved (Figure 3(H,J)), and TMEM119 expression was particularly abundant (Figure 3(J)). In the cortex of Case 2, CD68- and HLA-DR-positive activated macrophages were observed only in small numbers around the PrP plaques and rarely in the neuropil (Figure 4(A,C), inset: PrP plaque). In contrast, mild-to-moderate amounts of CD68- and HLA-DR-positive activated macrophages were observed in the white matter (Figure 4(B,D)). Iba1-positive microglia were mildly increased in the cortex and exhibited enlarged cytoplasm with thick processes (Figure 4(E), inset: PrP plaque). In the white matter, they moderately increased, and activated microglia with enlarged cytoplasm and thick processes were frequently observed (Figure 4(F), arrowheads). P2RY12-positive resident microglia in a homoeostatic state were present in small numbers in the cortex and white matter (Figure 4(G,H), inset: PrP plaque). In contrast, TMEM119-positive microglia were moderately preserved in the cortical neuropil, including around PrP plaques (Figure 4(I), inset: PrP plaque). Microglia around cotton-wool PrP plaques were positive for Iba1 and HLA-DR, but negative for CD68. The homoeostatic microglia marker TMEM119 was positive, whereas P2RY12 was absent. The expression of TMEM119 in microglia in the white matter was low (Figure 4(J)).

**Figure 5.**
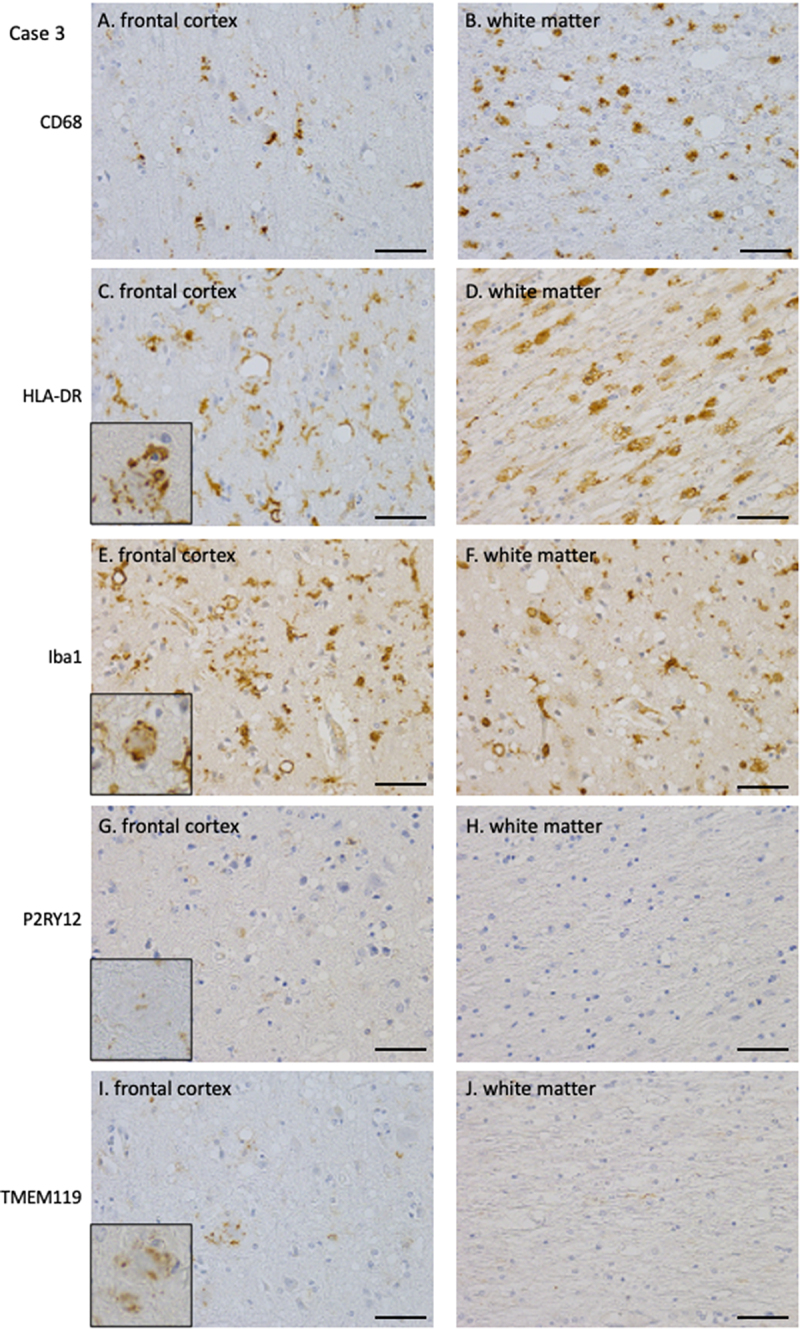
Immunohistochemical staining of microglia/macrophages in the frontal cortex and white matter of Case 3 (A, B: CD68, C, D: HLA-DR, E, F: Iba1, G, H: P2RY12, I, J: TMEM119, A, C, E, G, I: cortex, B, D, F, H, J: white matter) (A, B) CD68 immunohistochemistry demonstrated a mild to moderate presence of activated macrophages, predominantly in the white matter. (C, D) HLA-DR immunohistochemistry demonstrated numerous activated macrophages in the cortex and white matter, including around PrP plaques. (E, F) Iba1 immunohistochemistry demonstrates numerous activated microglia in the cortex and white matter, including around PrP plaques. (G, H) P2RY12 immunohistochemistry reveals a small number of positive microglia surrounding PrP plaques, with no positive cells detected in the cortical neuropil or white matter. (I, J) TMEM119 immunohistochemistry revealed a small number of positive microglia surrounding PrP plaques, with no positive cells detected in the cortical neuropil or white matter. PrP, prion protein.

The histopathological findings of microglia and macrophages in Cases 3–6 of P102L GSS, characterized by type-1 PrP^res^, 8-kDa PrP^res^, PrP plaques, and synaptic PrP deposits, are presented using Case 3 as an example. CD68-positive macrophages were observed in small amounts in the cortex and moderate amounts in the white matter (Figure 5(A,B)). Moderate numbers of HLA-DR-positive macrophages were observed in the cortex, including around the PrP plaques (Figure 5(C), inset: PrP plaque), and were abundantly present in the white matter (Figure 5(D)). Iba1-positive microglia/macrophages were observed in moderate to severe amounts in the cortex and white matter, including around the PrP plaques (Figure 5(E): cortex, 5F: white matter). Resident microglia, indicated by TMEM119-positive microglia, were observed only minimally around the PrP plaques (Fig. I, inset: PrP plaque) and was almost absent in the cortex and white matter, including those expressing P2RY12 (Figure 5(G-J)).

In summary, activated macrophages positive for CD68 or HLA-DR were less prominent in Cases 1 and 2 than in Cases 3–6. In contrast, homoeostatic markers of resident microglia, including P2RY12 and TMEM119, were observed in small amounts surrounding the PrP plaques in all cases. The presence of these markers in the cortical neuropil and white matter was restricted to Cases 1 and 2.

## Discussion

This study characterized the clinicopathological features of siblings with P102L mutation-associated GSS presenting exclusively with 8-kDa PrP^res^ (case 1:129 MM, haplotype: P102L-129 M; case 2:129 MV, haplotype: P102L-129 M). Both patients had an extended disease duration > 20 years. Pathological analyses revealed a predominance of amyloidogenic and non-amyloidogenic cotton-wool PrP plaques over synaptic PrP deposits. Despite the prolonged disease course, the spongiform changes and neuronal loss were mild to moderate. Moreover, microglial analysis demonstrated relative preservation of homoeostatic microglia.

In this study, Cases 1 and 2, who were siblings, demonstrated exclusive 8-kDa low-molecular-weight PrP^res^, without the presence of type-1 PrP^res^. Both patients had a history of medical interventions, with Case 1 receiving PPS treatment and Case 2 being treated with quinine hydrochloride and quinacrine. Both patients exhibited prolonged survival, exceeding 20 years; however, no clinical improvement associated with therapeutic intervention was observed [20]. Pathologically, Case 1 primarily exhibited amyloidogenic core-containing PrP plaques with only mild PrP synaptic deposits. Case 2 was characterized by predominantly non-amyloidogenic cotton-wool PrP plaques with mild to moderate PrP synaptic deposits. In both cases, synaptic PrP deposits were not prominent, and only 8-kDa PrP^res^ was detected via western blot analysis. In Case 2 and Case 5, the 8-kDa PrP^res^ signal detected with 3F4 was not demonstrable with either the N-terminal or C-terminal PrP antibodies. These findings suggest that the PK-resistant PrP of both cotton-wool and amyloidogenic PrP plaques lacks the *N*- and C-termini and is centred on the 3F4 epitope (aa 109–112). These findings suggest that, in addition to amyloidogenic PrP plaques, the main component of cotton-wool PrP plaques is aggregated PrP that is conformationally resistant to PK digestion and give rise to the 8 kDa PrP^res^ fragment. The presence of 8-kDa PrP^res^ as the main component of amyloidogenic PrP plaques has been reported [3]. Fluorescence and Congo red staining revealed that the cotton-wool PrP plaques were characterized by the absence of an amyloid core and the presence of fine clusters of spheroidal PrP. However, reports on the mechanisms underlying cotton-wool PrP plaque formation remain limited. In this study, the key difference identified between sibling cases from the same family was a codon 129 polymorphism. Case 2, which exhibited cotton-wool PrP plaques, was classified as P102L-129 MV, with the V allele located on the wild-type allele, opposite to the P102L mutation. Previous studies on P102L GSS have suggested that wild-type PrP, encoded with an allele opposite to the P102L mutation, contributes to abnormal PrP deposition [10]. Hill et al. reported two cases of GSS with P102L-129 MV (haplotype: P102L-129 M) (Table 4) [18]. One case (case 1756) demonstrated type-1 PrP^res^, with a disease duration of 9 months based on Wadsworth et al. [10], and was characterized by widespread spongiform changes and PrP amyloid plaques, which were identified differently from Case 2 in this study. Another case (case 3070) exhibited only 8-kDa PrP^res^, closely resembling the case presented here; however, histopathological findings were unavailable. Several reports of GSS cases with only 8-kDa PrP^res^ have indicated that amyloid PrP plaques are the predominant pathology, with minimal spongiform changes [15,21]. Piccardo et al. examined two cases exhibiting only 8-kDa PrP^res^: P102L-129 MM (haplotype: P102L-129 M, Case 2) and P102L-129 MV (haplotype: P102L-129 V, Case 8), and reported that these cases predominantly presented with amyloid PrP plaques without spongiform changes (Table 4) [21]. Parchi et al. examined two cases of P102L-129 MM (haplotype: P102L-129 M, Cases 6 and 7) exclusively exhibiting 8-kDa PrP^res^, and reported that these cases were also characterized predominantly by amyloid PrP plaques in the absence of spongiform changes (Table 4) [15]. Additionally, Young et al. reported a case of P102L-129VV GSS characterized by amyloid PrP plaques without spongiform changes and with a prolonged disease duration of 12 years, although the PrP^res^ pattern was unspecified (Table 4) [16]. These findings suggest a strong association between 8-kDa PrP^res^ and amyloid PrP plaques; however, they are insufficient to explain the pathomechanism underlying cotton-wool PrP plaque formation. A comprehensive analysis that takes in consideration the cases presented here suggests that the formation of cotton-wool PrP plaques may involve the combination of codon 129 M in the P102L mutant allele and codon 129 V derived from the normal allele, along with 8-kDa PrP^res^ as a critical contributing factor. Zahn et al. determined the nuclear magnetic resonance (NMR) structure of the intact recombinant human PrP [22]. In this report, PrP was described as comprising three α-helices and two β-strands, with β-strand 1 consisting residues 128–131. Omwansu et al. focused on the impact of the β-strand 1 including codon 129 polymorphism (residues 127–132) on the conversion to PrP^Sc^ and investigated structural changes associated with the M129 and V129 variants using molecular dynamics simulations combined with Markov state model analysis [23]. As a result, the PrP-M129 polymorph exhibited greater stability than PrP-V129, attributable to reduced random-coil motions, the formation of salt bridges, and an increased number of native contacts. Apostol et al. investigated, by means of crystallographic studies, 6-residue (127–132) PrP segments centred on residue 129—‘steric zippers’, defined as pairs of interacting β-sheets – in the context of codon 129 polymorphism [24]. They proposed that the heterozygous steric zipper model (M/V) is structurally less stable than the homozygous counterparts (M/M or V/V), thereby rendering the formation of an ordered array, such as an amyloid fibril, less favourable in the heterozygous condition. In light of these reports and our Case 2, the 129 V of the normal allele seems to play a pivotal role in the formation of cotton wool PrP plaques. However, Hill et al. reported PrP amyloid plaques in Case 1756, which exhibited only 8-kDa PrP^res^ and carried 129 V derived from the normal allele [18]. Therefore, in addition to the factors we consider critical for cotton-wool PrP plaques, other contributors may also play a significant role.

**Table 4.** Characteristics of cases exhibiting only 8-kDa PrPres and codon 129 polymorphism.

Case No.	Diagnosis	Codon 129	haplotype	PrPres	PrP plaque	synaptic PrP	spongiform change
1	GSS (P102L)	M/M	P102L-129 M	8kDa	amyloid PrP plaque	mild	mild
2	GSS (P102L)	M/V	P102L-129 M	8kDa	cotton wool PrP plaque	mild-moderate	mild-moderate
[18] case 1756	GSS (P102L)	M/V	P102L-129 M	type 1	amyloid PrP plaque	synapse +	widespread
[18] case 3070	GSS (P102L)	M/V	P102L-129 M	8kDa	NA	NA	NA
[20] case 2	GSS (P102L)	M/M	P102L-129 M	8kDa	amyloid PrP plaque	no	minimal
[20] case 8	GSS (P102L)	M/V	P102L-129 V	8kDa	amyloid PrP plaque	no	no
[15] case 6	GSS (P102L)	M/M	P102L-129 M	8kDa	amyloid PrP plaque	no	no
[15] case 7	GSS (P102L)	M/M	P102L-129 M	8kDa	amyloid PrP plaque	no	no
[16]	GSS (P102L)	V/V	P102L-129 V	NA	amyloid PrP plaque	no	no

kDa, kilodalton; L, leucine; M, methionine; V, valine; NA, not available; No, number; P, proline; PrPres, protease resistant prion protein.

Cotton-wool PrP plaques were identified in the cerebral cortex, hippocampus, and cerebellum. In contrast, these plaques were not observed in the putamen, globus pallidus, thalamus, or brainstem. The absence of detectable PrP deposits in the putamen, globus pallidus, and thalamus may be attributed to severe degeneration with a substantial loss of structural integrity in these regions, making the evaluation of PrP deposition challenging. Cotton-wool PrP plaques and limited amyloidogenic PrP cores have been identified in the cerebellum. These findings suggest that the formation of cotton-wool PrP and amyloidogenic PrP plaques may be influenced by codon 129 polymorphisms and region-specific microenvironmental factors in the brain. The tendency for amyloidogenic PrP plaque formation in the cerebellum has been reported and is a well-established finding.

In Cases 1 and 2, the synaptic PrP deposits were sparse, spongiform degeneration was mild, and CD68- and HLA-DR-positive activated macrophages were minimal. In contrast, in the other P102L GSS cases (Cases 3–6) exhibiting type-1 PrP^res^ and 8-kDa PrP^res^, P2RY12- and TMEM119-positive microglia in a homoeostatic state were almost absent, unlike those in Cases 1 and 2. The expression of P2RY12 and TMEM119, markers of homoeostatic microglia, decreases upon activating resident microglia [25–27]. Specifically, P2RY12 expression decreased rapidly, whereas TMEM119 expression remained relatively stable for a longer duration [25], which is consistent with the findings of this study. We previously demonstrated that in GPI-anchorless prion disease, which exhibits minimal spongiform changes, the activity of resident microglia is lower compared to that in P102L GSS and sCJD, with a greater retention of homoeostatic microglia. These findings suggest that microglial activation in prion diseases may be dependent on the PrP strain and on the presence of spongiform changes [28]. Collectively, these observations suggest that the primary component of PrP plaques and cotton-wool PrP plaques, 8-kDa PrP^res^, exhibits lower tissue pathogenicity than the primary component of synaptic PrP deposits, type-1 PrP^res^.

This study had some limitations. The total number of GSS cases analysed was limited to six, including two cases exclusively exhibiting 8-kDa PrP^res^ and one case of P102L-129 MV GSS with cotton-wool PrP plaques. Detailed analyses were performed; however, the small sample size limited our ability to fully elucidate the mechanisms underlying the exclusive presence of 8-kDa PrP^res^ and the precise formation process of cotton-wool PrP plaques. Therefore, the accumulation of additional cases in future studies is essential for gaining deeper insights.

## Conclusion

In P102L GSS cases exclusively exhibiting 8-kDa PrP^res^, PrP plaques and cotton-wool PrP plaques were predominant, whereas synaptic PrP deposits were minimally detected. Despite the prolonged survival observed in these cases, tissue damage remained mild. The principal components of PrP plaques may be 8-kDa PrP^res^, which might exhibit low tissue pathogenicity. Although speculative based on the present findings, the exclusive presence of 8-kDa PrP^res^ appears to be influenced by familial factors, with a potential contribution of epigenetic mechanisms; however, the precise underlying processes remain unknown. Furthermore, the formation of cotton-wool PrP plaques may require the involvement of both codon 129 V derived from the normal allele and 8-kDa PrP^res^.

## Materials and methods

### Participants

We investigated six autopsy GSS cases (P102L) at the National Hospital Organization Omuta Hospital and the Department of Neuropathology of Kyushu University. Table 1 shows the examined cases. Patient 1 was the older sister of Patient 2. Patients 1 and 2 had codon 129 MM (haplotype: P102L-129 M) and codon 129 MV, respectively. The V in case 2 was on the opposite allele of P102L (mutation haplotype: P102L-129 M). Case 1 initially presented with cerebellar ataxia as the first symptom, followed by dementia, and exhibited a phenotype consistent with classical GSS at 50 years of age. She received pentosan polysulfate sodium (PPS) infusion treatment 6 months after onset, but no clinical improvement was observed. Pentosan polysulfate was continuously administered at a dose of 120 mg/kg/day for approximately 14 months. The patient died of pneumonia 20 years after the onset of her illness. Patient 2 developed a right vestibular schwannoma at the age of 46 years and underwent tumour resection, during which a dural graft was implanted. Whether this was a Lyodura or not remains unknown. At the age of 53, she started to get lost on the way home from shopping, and other executive dysfunctions followed by psychiatric symptoms and gait disorders became evident. The patient exhibited a phenotype consistent with subacute dementia. She underwent treatment with quinine hydrochloride and quinacrine hydrochloride 6 years after onset, but no apparent therapeutic benefits were observed. Quinacrine hydrochloride was administered at a dose of 300 mg/day for a total duration of approximately four months, including temporary discontinuation due to hepatotoxicity. Quinine hydrochloride was administered for approximately one month but was also discontinued owing to hepatotoxicity. The patient died of pneumonia 25 years after the onset of her illness. Patients 3–6 had a prion genotype of P102L-129 MM (haplotype: P102L-129 M) with no familial relationship. None had a treatment history, and the duration of illness varied: 5, 10, 13, and 5 years, respectively.

### Molecular characterization of PrP

Brain samples (frontal cortex) were frozen at the time of autopsy and stored at −80°C until further use. The samples were homogenized to a final concentration of 10% in lysis buffer (100 mM Tris-HCl, 100 mM NaCl, 10 mM ethylendiamine tetraacetic acid, 0.5% Nonidet *p*-40, and 0.5% sodium deoxycholate, pH 7.6). To detect PrP^res^, the prepared homogenates were treated with 50 μg/mL PK for 1 h at 37°C. Samples were electrophoresed with sodium dodecyl sulphate polyacrylamide gel electrophoresis in preparative gels (Any kDa precast gel; BioRad, Hercules, CA) and transferred onto polyvinylidene difluoride membranes. PrP^c^ and PrP^res^ were detected using an anti-PrP antibodies (mouse monoclonal 3F4 [specific for PrP at 109–112, 1:400; BioLegend, San Diego, CA], mouse monoclonal 8B4 [specific for PrP at 37–44, 1:400, Santa Cruz Biotechnology, Dallas, TX], and rabbit monoclonal EP1802Y [specific for PrP at 212–230, 1:400, Abcam, Cambridge, UK]). Peroxidase-conjugated anti-mouse immunoglobulin G (AP192P, 1:3000; Chemicon, Tokyo, Japan) was used as the secondary antibody. Immunoreactions were visualized using an ECL Plus Western Blotting Detection System (GE Healthcare, Chalfont, UK).

### Neuropathological examinations

During the autopsy, the brains were fixed in 10% buffered formalin. The specimens included the middle frontal gyrus, precentral gyrus, superior and middle temporal gyri, inferior parietal lobule, anterior cingulate gyrus, amygdala, hippocampus, entorhinal cortex (at the level of the lateral geniculate body), calcarine cortex, basal ganglia, thalamus, cerebellum, midbrain, pons, and medulla oblongata. Paraffin-embedded tissues were cut into 6-μm sections and routinely stained. Haematoxylin and eosin, Klüver – Barrera, and Congo red staining were performed for histological examination.

Immunohistochemistry was performed using primary antibodies specific to anti-PrP (3F4, 1:200), anti-ionized calcium-binding adapter molecule 1 (Iba1) (rabbit polyclonal, 1:200; Wako, Osaka, Japan), anti-CD68 (mouse monoclonal CD68, 1:200, Dako, Santa Clara, CA), anti-CD163 (mouse monoclonal CD163, 1:200, Leica, Wetzlar, Germany), anti-HLA-DR (mouse monoclonal HLA-DR, 1:200, Dako), anti-transmembrane protein 119 (TMEM119) (rabbit polyclonal, 1:200, Sigma-Aldrich, St. Louis, MO), and anti-P2Y purinoceptor 12 (P2RY12) (rabbit polyclonal, 1:200, Sigma-Aldrich) as the primary antibodies. The sections were incubated overnight with primary antibodies at 4°C. Immunoreaction products were detected via polymer immunocomplex method using the Envision system (Dako) and the enhanced indirect immunoperoxidase method (Histofine Simple Stain MAX PO MULTI; Nichirei Biosciences, Tokyo, Japan). Nuclei were counterstained with haematoxylin. The immunoreactivity was visualized using 3,3′-diaminobenzidine (DAB; Dojindo, Kumamoto, Japan). Double immunofluorescence was performed using the following combination of antibodies: 3F4 and GFAP (rabbit polyclonal, 1:200, Dako). Alexa Fluor 488-labelled anti-rabbit immunoglobulin G (Invitrogen, Carlsbad, CA) and Alexa Fluor 546-labelled anti-mouse immunoglobulin G (Invitrogen) were used as secondary antibodies. Specimens were counterstained with 4′,6-diamidino-2-phenylindole (Invitrogen) and visualized using a Nikon A1R-A1 confocal microscopy system (Nikon, Tokyo, Japan).

## Data Availability

The data that support the findings of this study are available from the corresponding author upon reasonable request.
